# Investigating the impact of a research-based integrated curriculum on self-perceived research experiences of medical students in community placements: a pre- and post-test analysis of three student cohorts

**DOI:** 10.1186/1472-6920-14-161

**Published:** 2014-08-05

**Authors:** Judy R Mullan, Kathryn M Weston, Warren C Rich, Peter L McLennan

**Affiliations:** 1 Graduate School of Medicine, University of Wollongong, Northfields Ave, Wollongong, NSW 2522, Australia

**Keywords:** Research and critical analysis, Self-perceived research capacity, Medical students, Authentic learning experiences, Medical curriculum, Research projects

## Abstract

**Background:**

To build research capacity among graduating medical students, the teaching of research and critical analysis was integrated into the University of Wollongong (UoW) new, graduate-entry medical curriculum. This study examined whether the self-perceived research experiences of medical students, and consequent research capability, were influenced by exposure to this innovative research and critical analysis curriculum, which incorporated a 12-month community-based research project, and associated assessment tasks.

**Methods:**

The first three medical students cohorts (N = 221) completed a self-assessment of their research experiences in ten areas of research activity. Their responses were collected: before and after they undertook an individual community-based research project within a 12-month regional/rural clinical placement. The research areas investigated by the self-assessment tool were: (i) defining a research question/idea; (ii) writing a research protocol; (iii) finding relevant literature; (iv) critically reviewing the literature; (v) using quantitative research methods; (vi) using qualitative research methods; (vii) analysing and interpreting results; (viii) writing and presenting a research report; (ix) publishing results; and (x) applying for research funding.

**Results:**

Participation rates of 94% (207/221) pre-placement and 99% (219/221) post-placement were achieved from the three student cohorts. Following the successful completion of the research projects and their assessment tasks, the median responses were significantly higher (*p* < 0.05) in nine of the ten research areas. The only area of research for which there was no increase recorded for any one of the three cohorts, or overall, was (x) applying for research funding. This activity was not a component of the UoW research and critical analysis curriculum and the item was included as a test of internal validity. Significant gains were also seen between cohorts in some key research areas.

**Conclusions:**

Improved research capability among medical students was evidenced by increased scores in various areas of research experience in the context of successful completion of relevant assessment tasks. The results suggest that research capability of medical students can be positively influenced by the provision of a research-based integrated medical curriculum and further consolidated by authentic learning experiences, gained through conducting ‘hands-on’ research projects, under the supervision and mentoring of research-qualified academics.

## Background

The increasing international focus on building research capacity in primary health care [1] and the aspirations of the Australian Medical Council [2] to improve research understanding of graduating medical students has defined the need for medical schools to develop students’ research skills within their curricula. While this is often delivered through research electives, the *de novo* establishment of the Graduate School of Medicine (GSM) at the University of Wollongong (UoW) in 2007 provided the opportunity to embed the teaching of research and critical analysis (RCA) within the core curriculum.

The RCA curriculum is taught throughout the four-year graduate-entry medical degree program at UoW, which is based on an integrated Case-Based Learning (CBL) program and inclusive of the following four phases: phase one (an 18 month university-based phase); phase two (a 12 months hospital-based phase); phase three (a 12 month regional/rural community-based phase); and phase four (a six month advanced elective training and preparation for medical internship phase). In the first two phases of the teaching program, the RCA curriculum focuses on activities typical of the research paradigm [3], which include: developing skills in literature searching; critical appraisal; interpreting statistics; recognising different research methods and learning how to become evidence-based practitioners. These early aspects of the RCA curriculum are taught mainly as large group sessions, with RCA principles integrated into the context of the particular body system blocks that the students are being taught, as part of their integrated CBL program. For example: during the teaching of the ‘Gastrointestinal and Liver’ body system block, the *Helicobacter pylori* research conducted by Marshall and Warren [4] is used to discuss how evidence-based research can influence therapeutic guidelines; whereas during the ‘Cardiovascular and Respiratory’ body system block, students are taught how to interpret systematic reviews and meta-analyses by using the scientific evidence surrounding the use of statins for hypercholesterolaemia and the prevention of cardiovascular mortality. Thus, principles of evaluating evidence and understanding research design are embedded into the teaching of the body system blocks during the early phases of the medical program and the medical sciences are also embedded into the teaching of RCA in a two-way holistic approach to learning.

Active research opportunities are becoming more commonplace in a medical curriculum and typically include a dedicated research elective [5,6]. In keeping with the new, integrated UoW medical curriculum, as well as recommendations from the Australian Medical Council [2] to prepare and support student engagement in medical research, our challenges were to provide a research opportunity for every student and to integrate the research experience within their curriculum. This is achieved with the completion of a research project during their 12-month, phase 3 regional/rural community-based clinical placements [7], under the supervision and mentoring of a research-qualified UoW academic staff member. In an attempt to avoid students perceiving research as activities devoid of patient contact or relevance [8], our students are encouraged to undertake community-based research projects which are of personal interest to themselves. Based on evidence that students should be involved in the research activity from planning to execution [9], our students are required to identify and develop a research proposal, submit relevant ethics applications, collect and analyse data, write a journal-style final research report, prepare an abstract and present a conference-style poster to their academic supervisors and fellow students. Each of these requirements are summative assessment tasks, with the final journal-style report being marked independently by two assessors and their conference-style presentations being assessed by a research-qualified academic staff member.

In addition to the knowledge and understanding of the principles and skills underpinning RCA that were learned during the earlier phases of the integrated medical program, prior to the commencement of their research projects, students are provided with PowerPoint® online learning resources about research during this third phase. These PowerPoint® online resources, developed by the RCA team in response to the first cohort’s feedback about requiring more resources, include guides on: formulating a research proposal; undertaking a literature review; ethical considerations when undertaking human research; collecting and analysing quantitative and/or qualitative data; constructing research questionnaires and/or focus group/interview questions; writing a journal style research report; and writing a conference-style abstract and poster preparation. As these phase 3 students are widely dispersed geographically for their regional/rural community-based placements, each is provided with individual supervision and mentoring by the allocation of a research-qualified and experienced UoW academic staff member. Supervision is largely conducted by email, teleconference and/or videoconference and, in some cases, primary care preceptors and/or experienced local supervisors also choose to collaborate with the students on the research project.

Millar *et al.*[10] report that many graduating doctors believe their knowledge of basic research skills is poor because research is not taught in the medical curriculum and subsequently it is lacking in professional practice. The common inclusion of critical thinking and research training amongst professional development modules delivered by professional colleges recognises this need in practising doctors [10]. The integrated GSM RCA curriculum is, therefore, designed to address this situation by ensuring that graduates have the knowledge and experience required to incorporate the requisite RCA skills into their everyday practice. Based on the studies undertaken by Vygotsky [11] in ‘meta-cognition’ and ‘self-regulated learning’, the UoW medical students are encouraged to create their own area of inquiry, affording them the opportunity to be exposed to ‘situated learning’ [12] and thus allowing them to further develop a deep learning and understanding [13] about research and critical appraisal. Furthermore, engagement in a research project of their own choice that deals with real world problems [14] helps to foster ‘authentic’ learning [15,16] and motivation [17] because students view the task as relevant and interesting. Then, as the research project builds upon and incorporates the RCA principles learnt in earlier phases of the program, the project becomes a conduit for ‘meaningful learning’ [18]. This is where “learning can be related to previous knowledge and related to a pre-existing cognitive framework” [18] p.201].

In addition, the phase 3 regional/rural community-based research project fulfils Harden & Laidlaw’s [19]**FAIR** model (**F**eedback**, A**ctivity, **I**ndividualisation, **R**elevance), which leads to more effective learning. Through the use of research-trained and experienced academic supervisors, students receive regular, timely and supportive feedback throughout all stages of their research project. Importantly, the project is undertaken by the student themselves, making them the active learner/researcher while being guided by their supervisors. The supervisors support the students in designing their own research projects, around the research question they wish to answer, making each project individual and unique. Finally, the research project is relevant to the students according to both the professional requirements of the Australian Medical Council [2] and their own personal interests, providing them with the opportunities to participate in research activities and disseminate their findings, after graduating from university; an opportunity, which many medical students have not had in the past [20,21].

This study explored medical student research capability, as measured by self-assessed levels of research experience in conjunction with the successful completion of RCA summative assessment tasks. Furthermore, the study investigated whether such research capability was influenced by student exposure to the integrated RCA curriculum, which incorporated a 12-month regional/rural community-based research project as part of their phase 3 clinical placements.

## Methods

Human research ethics approval was granted by the UoW Human Research Ethics Committee (HREC). Participants in the research were the first three UoW medical student cohorts (N = 221), cohort 1 (n = 68) graduated in 2010, Cohort 2 (n = 76) graduated in 2011 and Cohort 3 (n = 77) graduated in 2012. Their research capabilities were established using their self-reported score for research experience as assessed by the ‘research spider’ tool [22], in the context of concurrent successful completion of the following research-related summative assessment tasks: the development of a research proposal; completion of university human research ethics requirements; development of a research tool; collection and analysis of research data; completion of a journal-style report; and the preparation and presentation of a conference-style poster to peers and academics. The ‘research spider’ assessment tool [22] was administered to each student prior to, and at the completion of, their 12-month regional/rural community-based clinical placement, which commenced 2.5 years into their 4-year medical degree program. This tool asked students to indicate their level of research experience using a 5-point Likert scale (1 no experience; 5 very experienced) investigating ten areas of research (See List of Research Experiences Assessed).

### Research experiences assessed

i. defining a research question/idea

ii. writing a research protocol

iii. finding relevant literature

iv. critically reviewing literature

v. using quantitative research methods

vi. using qualitative research methods

vii. analysing and interpreting results

viii. writing and presenting a research report

ix. publishing results

x. applying for research funding

The option (x) applying for research funding, served as an internal test of the assessment tool’s validity [23] because this research skill is not taught as part of the RCA curriculum. In cases where students did not return a response to one particular item of the ‘spider’, the pre- or post- ‘pair’ to that particular response was omitted from “within cohort” pre- to post- analysis. Data are presented as the percentage of students responding at each score, or median values for their responses. The differences between median values, pre- and post-placement, were tested using the non-parametric Wilcoxon rank sum test and differences between cohorts in the proportion of responses, above and below the median, were tested using the non-parametric two sample median test (*χ*^2^). Within cohorts, individual item mean scores were tested using a paired *t*-test (Statistix 8, Analytical Software, Tallahassee, FL, USA). Significance was accepted at *p* < 0.05.

## Results

### Response rate

All UoW GSM students successfully completing their phase 3 regional/rural community-based clinical placements were eligible for inclusion in the study (N = 221). The overall pre-placement response rate was 94% (207/221) (Cohort 1, n = 68; Cohort 2, n = 70; Cohort 3, n = 69); and the post-placement response rate was 99% (219/221) (Cohort 1, n = 68; Cohort 2, n = 76; Cohort 3, n = 75). All medical students included in the analysis successfully completed the required summative research-related assessment tasks by the end of the research period.

### Comparison of research scores before and after the research project experience

**Figure 1:**
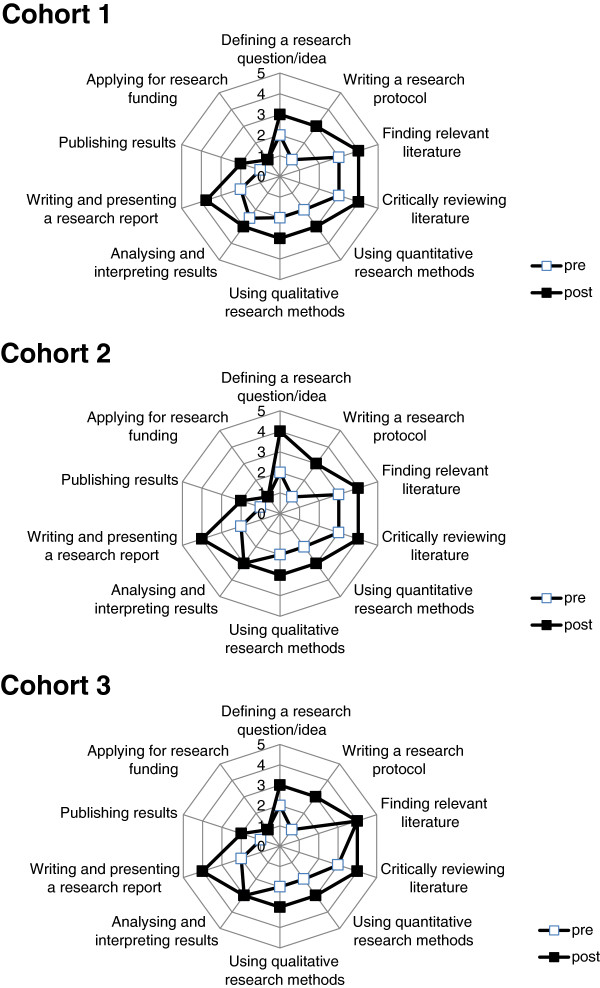
Research experience scores of consecutive medical student cohorts: pre- and post- placement.

**Figure 2:**
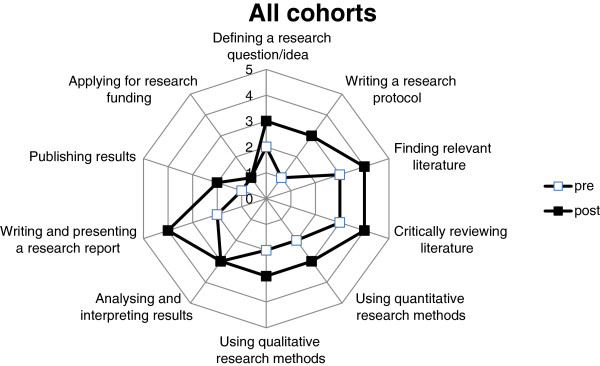
Combined research experience scores of three consecutive medical student cohorts: pre- and post-placement.

The median scores for the research items investigated on completion of the student projects was significantly higher than at commencement in nine of the ten research areas evaluated within each cohort (Figure 1) and in total (Figure 2). These areas were: (i) defining a research question/idea; (ii) writing a research protocol; (iii) finding relevant literature; (iv) critically reviewing literature; (v) using quantitative research methods; (vi) using qualitative research methods; (vii) analysing and interpreting results; (viii) writing and presenting a research report and (ix) publishing results. All research areas demonstrated an increase in the proportion of responses above and below the combined median, both within cohorts and overall (*p < 0.0001*). The only research area for which there was no increase in score recorded for any of the three cohorts, or overall, was (x) applying for research funding.

The results also indicate that the mean scores for individual students were significantly higher on completion of the 12-month placement (mean (95% CI): 3.09 (3.00 – 3.18), than at the commencement of the placement (2.23 (2.13 – 2.33) (paired *t*-test, p < 0.0001).

**Figure 3:**
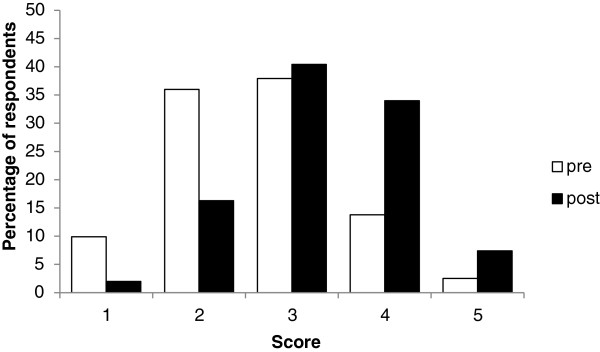
(vii) Analysing and interpreting results: Combined pre- and post-placement scores of all three medical student cohorts.

**Figure 4:**
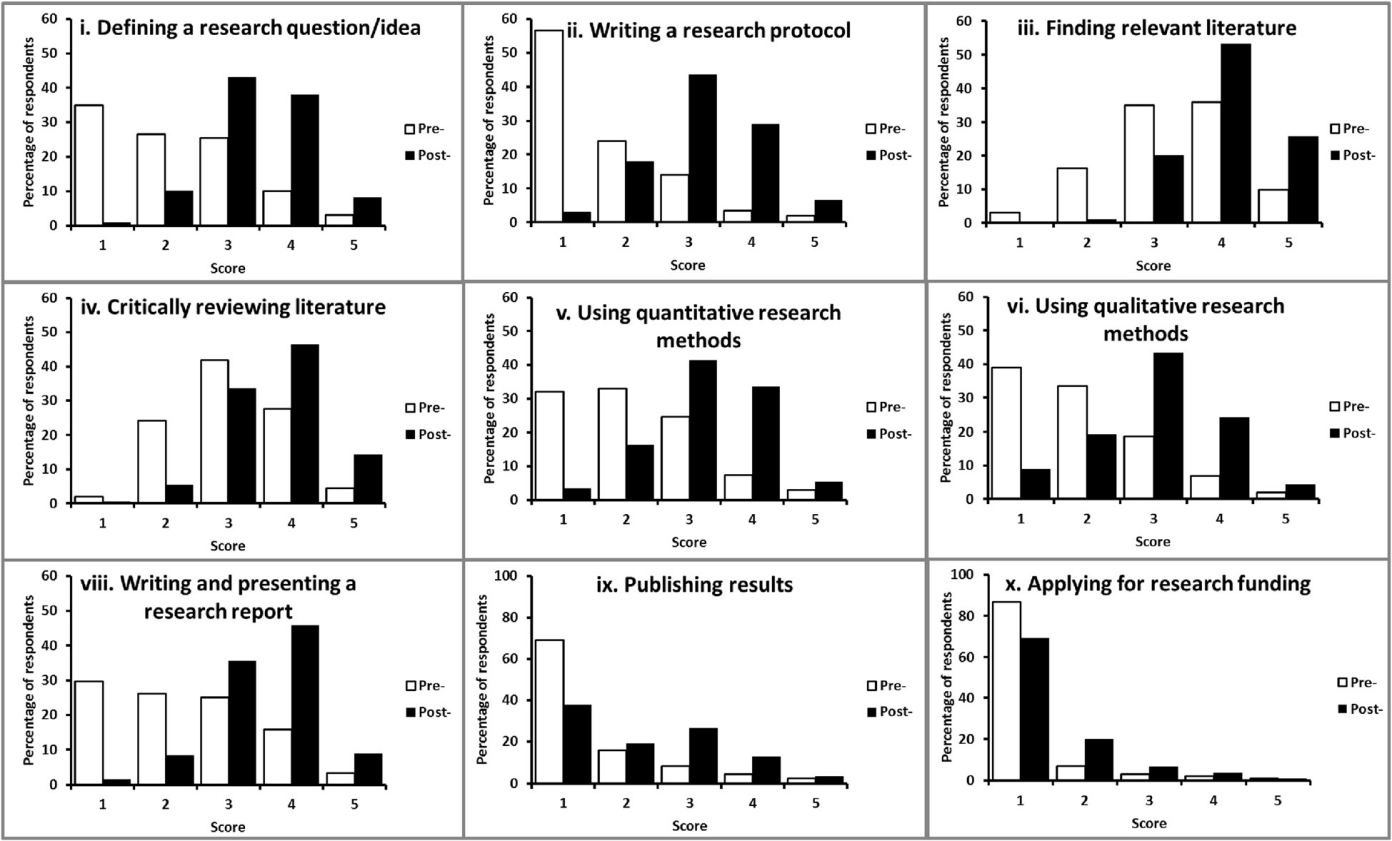
**Areas of research: Combined pre- and post-placement scores for all three medical student cohorts.** Numbering refers to the list of Research Experiences Assessed: i. defining a research question/idea; ii. writing a research protocol; iii. finding relevant literature; iv. critically reviewing literature; v. using quantitative research methods; vi. using qualitative research methods; vii. analysing and interpreting results; viii. writing and presenting a research report; ix. publishing results; x. applying for research funding. N = 207.

Within each cohort, the largest increases in scores from pre- to post-placement were found in (ii) writing a research proposal and (viii) writing and presenting a research report. Within the research area of (vii) analysing and interpreting results (Figure 3), outcomes at completion of the placement were significantly different to the outcomes at commencement, despite recording a response median of 3 in both pre- and post-placement surveys. For this research area, the proportion of responses above the median at completion of the placement (82 above; 38 below; with 83 ties), was significantly greater than at commencement (34 above; 97 below; with 77 ties) (*p < 0.0001*). This is illustrated as a rightward shift in the post-placement responses (Figure 3). Similar rightward shifts and significant improvements in research scores were also apparent in each of the other research areas, except for (x) applying for research funding (Figure 4).

### Comparison of research scores across the three successive medical student cohorts

Comparison of all pre-placement responses showed a significant increase across the successive cohorts (i.e. improvement sequentially from Cohort 1 to Cohort 3) in their responses to (iv) critically reviewing literature (*p = 0.002* for slope), with a non-significant trend for (v) using quantitative research methods (*p = 0.08*). An individual between-cohort comparison showed a significant increase in proportions above the median for (iii) finding relevant literature from Cohort 2 to Cohort 3 (*p = 0.025*) and from Cohort 1 to Cohort 3 (*p = 0.002*).

Comparison of all post-placement survey responses showed a significant increase successively across the cohorts (from 1 to 3) in their responses to: (iv) critically reviewing literature (*p = 0.025* for slope); (v) using quantitative research methods (*p = 0.013*); and (vii) analysing and interpreting results (*p = 0.036*), with non-significant trends for (i) defining a research question/idea (*p = 0.057*), (ii) writing a research protocol (*p = 0.10*) and (iii) finding relevant literature (*p = 0.081*). An individual between-cohort comparison showed significant increases in proportions above the median from Cohort 1 to 2 for (v) using quantitative research (*p = 0.025*) and (vii) analysing and interpreting results (*p = 0.022*), and from Cohort 1 to 3 for (i) defining a research question/idea (*p = 0.040*), (ii) writing a research protocol (*p = 0.023*), (v) using quantitative research methods (*p = 0.026*) and (vii) analysing and interpreting results (*p = 0.046*), with a non-significant trend for (iii) finding relevant literature (*p = 0.11*).

To date, findings from ten of the student projects have been published in peer-reviewed journals, or are on a publication pathway, and/or have been presented at either national or international conferences. These additional findings, which were not part of the original study goals, provide strong evidence that the program is effective.

## Discussion

Research training is widely recognised as an important component of a medical curriculum [24,25] and involving students in the practicalities of research can be instrumental in developing a sound appreciation and understanding of research [9]. This study investigated the influence of a research-based curriculum and hands-on research experience in the development of medical student research capability. Our interpretation of increased research capability was made after assessing the student scores in ten research areas represented in the ‘research spider’ self-assessment tool [22] in the context of their successful completion of various research-based assessment tasks by the end of the research period.

Our study provides evidence that research capability of medical students can be significantly improved when students exposed to a research-based integrated curriculum are provided with the opportunity to conduct a research project. The contribution of experienced academic staff providing key supervision, mentoring and direction throughout the process, is essential in this context [9].

As a result of this research component of their integrated medical degree program, the students in the current study were more capable in: writing a research protocol; finding relevant literature; using and analysing quantitative and/or qualitative data collection methods; and writing a journal-style publication and/or conference-style presentation. The lack of improvement in their research experience scores for (x) applying for research funding confirms its use as an internal control to establish the validity of the survey results [23] because it was not taught as part of the research-based integrated curriculum. The internal validity of our study was further protected by conducting the pre- and post-placement surveys more than ten months apart, with students blinded to their prior responses.

The significant improvement sequentially with each new cohort (i.e. from Cohort 1 to 3) in their post-placement responses for (iv) critically reviewing literature, (v) using quantitative research methods and (vii) analysing and interpreting results, coincides with the RCA team’s development and refinement of the aforementioned phase 3 PowerPoint® online resources made available to the students in response to the perceived need of Cohort 1. Similarly, the significant improvement of pre-placement responses across the cohorts (from 1 to 3) in (iv) critically reviewing the literature, may reflect a major revision of the preliminary RCA curriculum after the first cohort of students had completed phase 1. During these revisions, the earlier phases of the RCA curriculum were integrated to focus on evidence-based medicine and activities typical of the research paradigm [3], rather than exposing the students to independent instruction in practical statistics and scientific methods. It could also be argued that the other trends towards improvement in perceived research capabilities across the three cohorts were positively impacted upon by the students’ exposure to experiential learning achieved by undertaking their individual research projects.

As an overall strategy to engage the students actively in research, our results suggest that participation in a research project during their medical course provides an opportunity for future medical practitioners to develop and improve their research capabilities and that a research-based integrated curriculum can be directed at preparing for and improving this experience. Most of the student projects were limited in scope, which was a deliberate approach based on evidence [26,27] that individuals need to slowly build their research capacity by starting off with small scale studies investigating useful, practice-based problems to gain the experience and confidence to successfully continue with future research projects. This practice may redress concerns [10] that many graduating doctors do not feel they are suitably taught or shown how to undertake research. While it is beyond the scope of our study to assess whether the medical students will participate in future research, other studies have indicated that medical practitioners who have been exposed to research education are more likely to undertake research in their practice [28-30].

For all three cohorts in our study, the biggest perceived improvements noted over the course of undertaking their research projects were in (ii) writing a research protocol and (viii) writing and presenting a research report. These improvements likely reflect the impact of the provision of relevant online research resources, as well as the individual mentoring and supervision they received from the research-qualified academic staff within the GSM in augmenting the students’ own experiences. Furthermore, the improvements are evidence that incorporating these particular tasks as assessable components of the degree program help to improve student learning about how to undertake and report on research findings. Moreover, the accepted peer-reviewed journal publications, as well as the national/international conference presentations based on the students’ projects, to date, demonstrate the added value of the mentoring and feedback that the students receive, encouraging students’ confidence in conducting research, as well as in publishing and disseminating their findings. This is arguably the key to building research capacity [31]. Furthermore, by transitioning from being a passive learner (in phases 1 and 2) to an active learner (in phase 3), students developed these research capabilities through the construction of their own knowledge derived from interactions amongst themselves, the context of their project and what they have previously learnt. Essentially, by undertaking their own research projects, students become involved in a more holistic integrated approach to their learning, rather than experiencing an ‘atomised’ approach where learning is broken into individual ‘modules’ [14].

Crucial to the students’ transition from passive learning to an active construction of personal knowledge is the design of the UoW RCA integrated curriculum, which provides a learning experience that is authentic to the student and not artificial. Authentic learning tasks are “genuine and embedded in real life” [14] p.716] and reflect the active participatory experience of learning [32], as opposed to the traditional passive transmission model. To be authentic, the tasks need to have connection to real-life problems faced by the learner and to involve critical thinking and synthesis of knowledge, with the outcomes being relevant to the learner’s context [33]. These elements are core to the UoW RCA integrated curriculum and to the research projects undertaken by the students, which mainly focus on community-based health issues.

The research topics chosen by our students in the present study were varied and used quantitative, qualitative and mixed-method research study designs. The topics included patient-oriented, practitioner-oriented and community-wide interests encompassing clinical audits, patient surveys and even practitioner surveys and lifestyle interventions. With the phase 3 community-based clinical placements being conducted in regional and rural areas within Australia, the research topics often focused on public health issues relevant to these communities. The added benefits of researching such topics is an increased awareness about health issues experienced by these communities and improved opportunities for engagement in research by the regional and rural practitioners themselves [34,35]. The end result may be the establishment of groups of like-minded researchers [36], with similar interests in topics relevant to the practitioners and the community [26]. Future studies should therefore focus on developing a more integrated approach to developing student/practitioner research skills, which has the potential to increase overall research capacity in primary health care [27,36].

While it is recognised that self-assessment tools have their limitations [37,38] the results presented in this study display considerable rigor in demonstrating increased research capability among medical students. The study was highly reproducible, with this paper describing the same level of statistically significant change in three successive cohorts of medical students, each of which was surveyed pre- and post- their 12-month research/placement experience, without any reference to their prior responses or to the outcomes of previous cohorts. Moreover, the students were not specifically trained or coached in any of the research spider tasks during their phase 3 placements. The significant difference between median and mean scores, pre- and post-, is well beyond the previously validated test-retest reliability of the instrument [22]. The impact of the research project on the robustness of our findings about improved students’ perceptions regarding their research capabilities is further demonstrated by the successful completion and assessment of individual journal-style research reports and conference-style presentations, as well as the success of some students in having their work published and/or presented at national/international conferences. We can therefore be confident that the differences observed are due to the authentic research experiences gained by the students and being exposed to an integrated RCA curriculum, rather than being due to increased experience in using the ‘spider’ tool.

Our results are also supported by those originally published by Smith *et al. *[22], who showed in a cross-sectional analysis that the ‘spider’ results correlated with actual research experience. In our study, the mean score of our students at baseline was above that of Smith *et al.*’s mean result for primary care physicians with no research grant or publication experience. This likely represents that Smith *et al.*’s primary care physicians, of 15 years ago, would have had little or no research skills built into their medical training. The relatively high scores of our medical students suggest that the inclusion of research and critical analysis as an integrated component throughout the medical curriculum, including conducting a research project, is influential and important in developing research-ready medical practitioners.

## Conclusion

Our study provides evidence that the authentic learning experience of conducting supervised research projects, as part of an integrated research and critical analysis curriculum, within a medical school program, can significantly improve medical students’ self-perceived research experiences. Moreover, in association with successfully completing assessment tasks that are also research-related, the data provide evidence for a positive impact on the students’ research capability. Research skills and experience can clearly be acquired in a regional or rural setting, integrated within a community placement program, without entree to the vast resources required to provide traditional research exposure by intense elective or immersion in a dedicated research laboratory. Importantly, unlike the narrow expectations of a research elective where students may participate in limited components of an existing project, this study shows the value of exposure across all components of the research process from defining a research question to completing a final report, thereby delivering a comprehensive research experience.

## Abbreviations

GSM: Graduate School of Medicine; RCA: Research and critical appraisal; UoW: University of Wollongong.

## Competing interest

The authors report no competing interest and are responsible for the content and the writing of this article.

## Authors’ contribution

JM, KW, WR and PMcL were all responsible for collecting and analysing the data for this research project and were all involved in writing the manuscript. All authors read and approved the final manuscript.

## Authors’ information

1. Dr JR Mullan PhD is senior lecturer in medical sciences and theme leader for research and critical analysis at the University of Wollongong, Graduate School of Medicine, Wollongong, NSW 2522 Australia

2. Dr KM Weston PhD is senior lecturer in public health at the University of Wollongong, Graduate School of Medicine, Wollongong, NSW 2522 Australia

3. Dr WC Rich ScEdD is lecturer in research and critical analysis University of Wollongong, Graduate School of Medicine, Wollongong, NSW 2522 Australia

4. Professor PL McLennan PhD is professor of physiology and faculty research chair at the University of Wollongong, Graduate School of Medicine, Wollongong, NSW 2522 Australia.

## Pre-publication history

The pre-publication history for this paper can be accessed here:

http://www.biomedcentral.com/1472-6920/14/161/prepub
